
JOURNAL INFORMATION
==============================
NLM Title Abbreviation: ScientificWorldJournal
Journal ID: ScientificWorldJournal
NLM Journal ID: 101131163
Journal ID: TSWJ

The Scientific World Journal

ISSN: 2356-6140
EISSN: 1537-744X
Publisher: Wiley

ARTICLE INFORMATION
==============================
PMCID: PMC5956486
PMID: 15349533
DOI: 10.1100/tsw.2004.52
Article ID: 352856
Article version: 1
Subjects: Original Report

Penile Fracture and Associated Urethral Injury

De Stefani S. *
Stubinski R.
Ferneti F.
Simonato A.
Carmignani G.
Department of Urology, University of Trieste, Italy
*Corresponding author.Academic Editor: Anthony Atala

Publication date: 2004
Electronic publication date: 2004 Jun 7
Volume: 4
First page: 92
Last page: 99
Copyright: Copyright © 2004 S. De Stefani et al.
Copyright year: 2004
License: This is an open access article distributed under the Creative Commons Attribution License, which permits unrestricted use, distribution, and reproduction in any medium, provided the original work is properly cited.
License URL: https://creativecommons.org/licenses/by/3.0/

==============================
Original Report
*TheScientificWorldJOURNAL* (2004) 4 (S1), 92–99
ISSN 1537-744X; DOI 10.1100/tsw.2004.52

### Penile Fracture and Associated Urethral Injury

S. De Stefani*, R. Stubinski, F. Ferneti, A. Simonato, and G. Carmignani
**Department of Urology, University of Trieste (Italy); Department of Urology, University of Genoa (Italy)*

*Previously published in the Digital Urology Journal*

**DOMAIN**: urology

Fracture of the penis is an uncommon pathology[1]. It consists in a rupture of the tunica albuginea of one or both corpora cavernosa following injury to an erect penis. The most common causes are blunt trauma during sexual intercourse, masturbation, unconscious nocturnal penile manipulation or a fall onto the erect penis[7]. The reason why rupture of the albuginea occurs only during erection lies in the fact that in the normal flaccid condition the penis occupies a position which is well protected against blows or blunt traumas. In the erect penis the tunica albuginea thins from 2 mm to 0.5 - 0.25 mm and thus it is more susceptible to traumatic tearing. Penile fracture associated with urethral injury is even more uncommon and accounts for 10 to 20% of reported cases. Prompt diagnosis and immediate surgical repair allows for earlier resumption of sexual activity and gives a lower incidence of penile chordee secondary to blood clot absorption and fibrous tissue formation[2]. Surgery is mandatory for the prevention of late sequelae following injury especially in cases associated with urethral rupture. At times the corporeal tear is very large and in patients with concomitant lesions involving the urethra surgical repair can become difficult and require particular technical shrewdness.

We report the surgical repair of 8 cases of penile fracture, two of which were complex involving both corpora cavernosa and an associated partial rupture of the urethra.

### MATERIAL AND METHODS

Between 1986 and 1997, 8 cases of blunt penile trauma were referred to our institution. Patients were admitted to emergency room between 2 and 48 hours following the accident. History revealed that sexual intercourse was the commonest ethiology (5 cases), followed by masturbation (2 cases) and a fall onto the erect penis as a consequence of heavy alcohol intake in one patient.

All patients refer to have heard a characteristic "cracking sound" during intercourse followed by immediate loss of erection, onset of severe pain and swelling of the penis. The subsequent penile hematoma which formed, brought about penile deformity and discoloration, which differed in severity from patient to patient. In one patient blood loss from the external urethral meatus was present (**Fig.1**) and another patient complained of the inability to urinate from the moment of the accident onwards (which occurred 12 h beforehand). In 4 cases the hematoma and shaft deformity were so severe that the patients immediately underwent explorative surgery without any further investigation (**Fig.2**). In the remaining 4 patients, presenting a more circumscribed hematoma, the site of injury was evidenced by means of

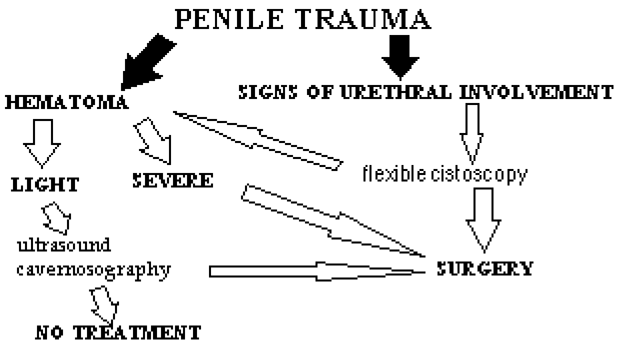

**FIGURE 1A.** Flow-chart for surgical treatment of penile trauma.

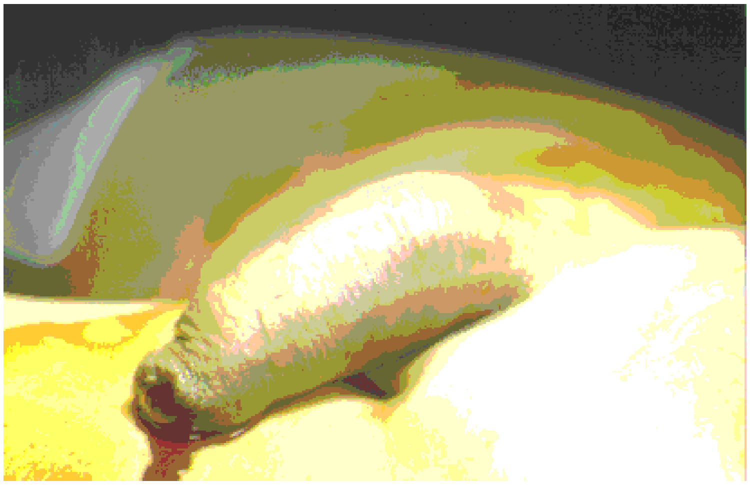

**FIGURE 1**. In case of blood coming out from the urethra an urethral lesion should be always suspected even in case of limited hematoma and slight penile swelling.

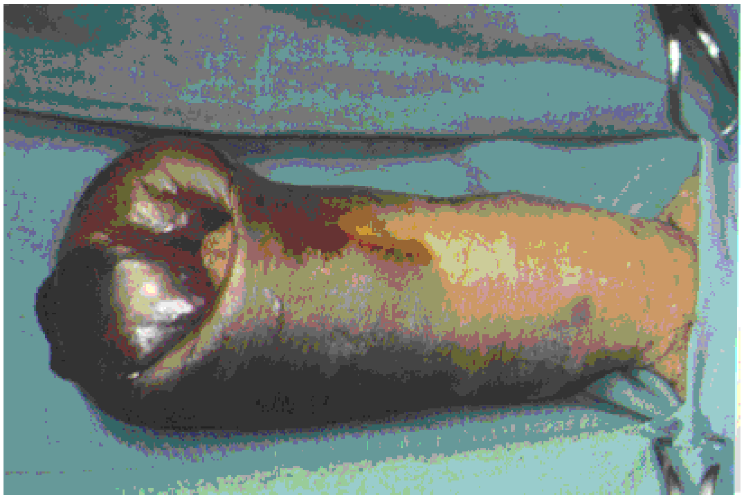

**FIGURE 2.** Penile swelling and hematoma are evident even if the extent of the lesion in corpora cavernosa is not always comparable to physical signs.

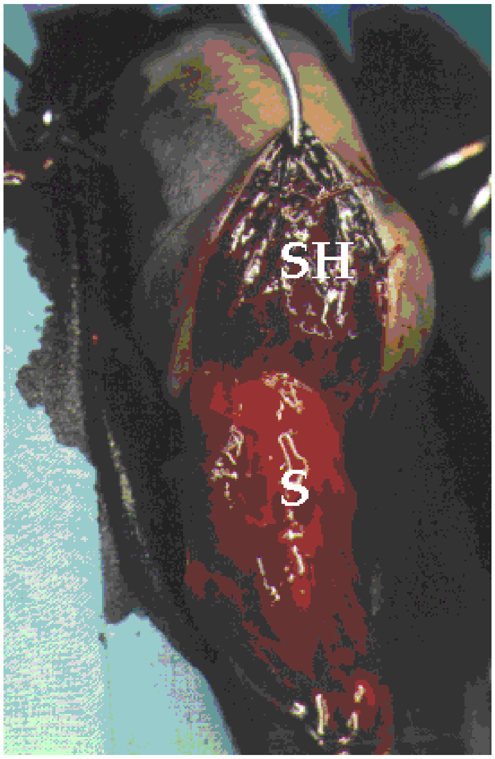

**FIGURE 3**. Shaft (S) and subcutaneous hematoma (SH) are evident after penile degloving.

contrast enhanced cavernosography. We were not able to identify the site of injury by simply palpating the penis, as has been reported in the literature by others authors, in any of our patients. In two cases the lesion of albuginea was clearly visible during an ultrasound examination.(**Fig.8**) Two patients, one with a severe hematoma and one with a more limited lesion, also had a urethral rupture. In both flexible cistoscopy was performed immediately before surgery, and the urethral lesion was evidenced.

Operation was performed via a circumferential subcoronal incision. After evacuation of the hematoma (**Fig.3**) the corporeal tear was identified (**Fig.4**) and closed with a 4/0 PDS interrupted suture. In one case the lesion was very large and reconstruction of the corpora was carried out by means of a split thickness dermal graft harvested from the left thigh (**Fig.5**).

In two cases the corpus spongiosum and urethra were partially severed (**Fig.6**). Reconstruction was performed using a 6/0 polyglactin suture after debridment of the wound edges. In one case the urethral lesion was almost complete and located at the level of the corpora cavernosa tear. After urethral repair, a subdartoic vascularised blanket flap was wrapped around the urethra to improve its healing and separate the urethra from the albuginea at the site of the lesion.

A 14 Fr. urethral Foley catheter was placed during surgery in order to prevent inadvertent urethral damage (except in cases in which a urethral injury was suspected) and removed 24 hours after the operation. In those patients in which the urethra was reconstructed, the catheter was removed after 7 days. Perioperative antibiotic prophylaxis was instituted in all cases, our preference was a third generation cephalosporin

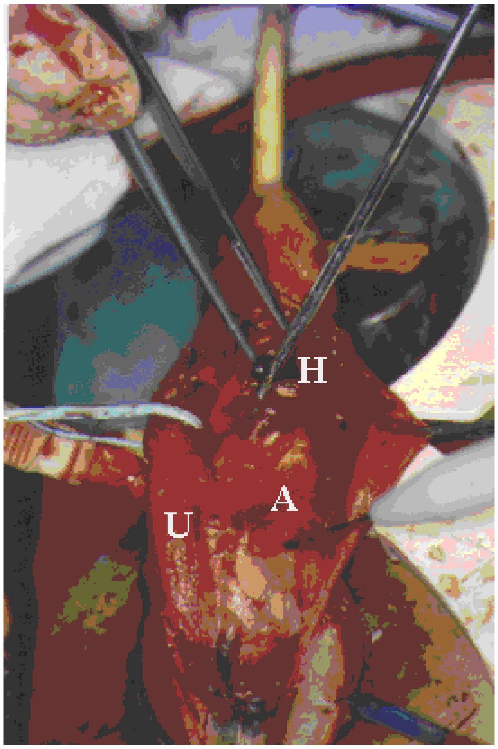

**FIGURE 4.** Penile shaft is degloved and Buckís fascia is entered to expose the hole (H) in the albuginea (A). The urethra (U) is isolated and pulled apart by means of a coloured rubber band.

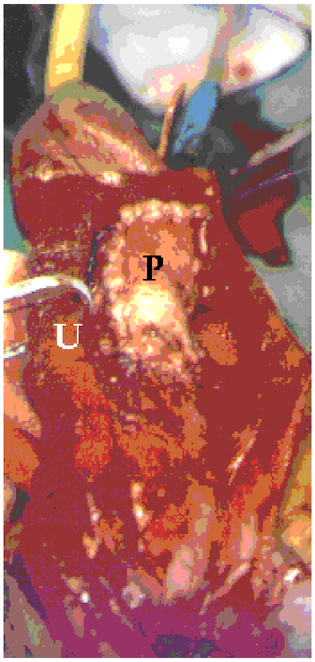

**FIGURE 5.** In this case the lesion was very large and reconstruction of the corpora was carried out by means of a split thickness dermal graft (P) harvested from the left thigh.

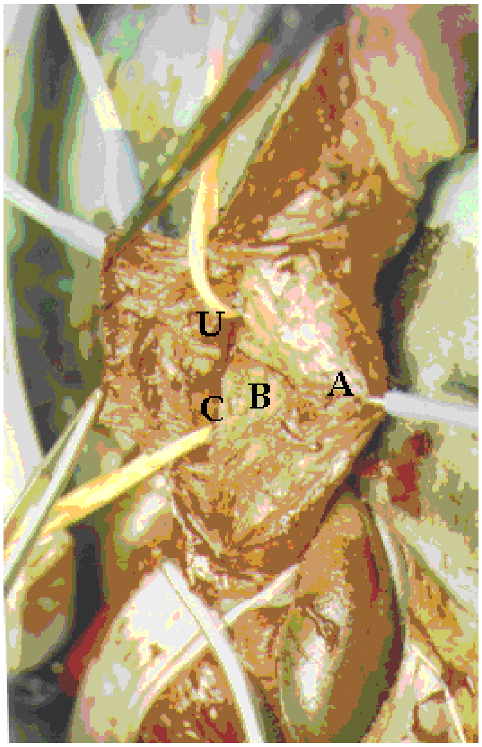

**FIGURE 6**. Operating field and Scottís retractor. In this picture are clearly visible: the albuginea (A) which is retracted laterally by means of an hook; the site of corporeal rupture (B); the urethra (U) and its site of rupture where the catether (C) is evident.

.

### RESULTS

Follow-up ranged from 2 months to 8 years (average 3.7 years). There were no significant postoperative complications and the patients were discharged from the hospital 3 days following operation. Six patients have a normally conformed penis on erection with optimal functional and aesthetic result whilst two presented a mild curvature due to residual fibrosis (**Fig.7**). These two patients underwent surgery 24 and 48 hours after the trauma, one has a follow-up which is too short to be significant (2 months). All patients were potent as evidenced by means of pharmacological (prostaglandin) induced erection and patient questioning. Urethrography and flexible cistoscopy demonstrated an excellent urethral healing at 8 months and 1 year follow-up after urethral reconstruction.(**Table2**)

### DISCUSSION AND CONCLUSION

In a literature review approximately 200 cases of penile fracture have been reported and 10% of these had an associated urethral injury. Diagnosis is generally made from the history and physical examination. However, at times it may be difficult especially if urethral injury is not suspected or the penile hematoma

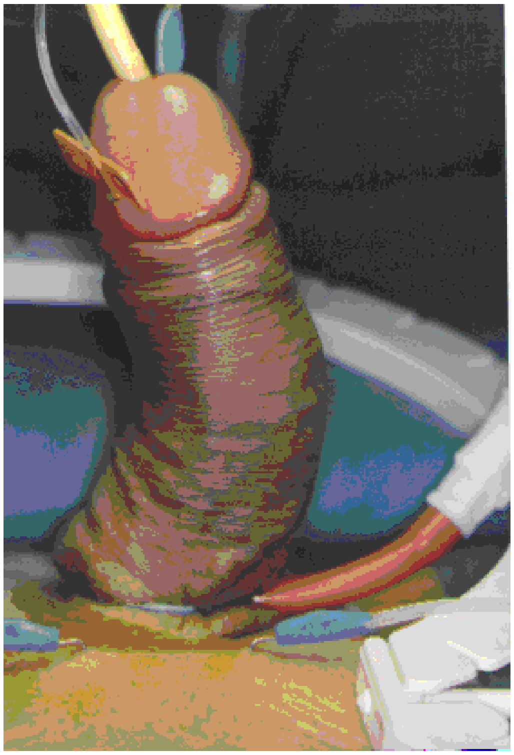

**FIGURE 7.** Penile curvature due to postoperative fibrosis is present one years after the operation. The patient was operated 48 h. after trauma.

**TABLE 2**

| Pt | Age | Hemotoma | Voiding Problems | Elapsed Time | Cystoscopy | First Approach | Urethral lesion | Follow-up | Healing |
|---|---|---|---|---|---|---|---|---|---|
| 1 | 22 | severe | no | 2 hours | intraoperative | surgery | no | 18 months | ok |
| 2 | 20 | severe | no | 10 | intraoperative | surgery | no | 8 years | ok |
| 3 | 35 | severe | no | 6 | intraoperative | surgery | no | 28 months | ok |
| 4 | 31 | severe | blood from urethra | 48 | preoperative | surgery | yes | 12 months | curvature |
| 5 | 20 | light | yes | 12 | preoperative | ultrasound + cav | yes | 8 months | ok |
| 6 | 43 | light | no | 5 | no | cavernosography | no | 5 years | ok |
| 7 | 25 | light | no | 12 | no | ultrasound + cav | no | 12 months | ok |
| 8 | 30 | light | no | 24 | no | cavernosography | no | 2 months | curvature |

is limited, because hematoma is not pathognomonic for corpora cavernosa rupture. Preoperative cavernosography must be strongly recommended in cases with limited hematoma or penile skin ecchymosis because the extent of the lesion in corpora cavernosa is not always comparable to physical signs, as mentioned above. In fact we observe done case of urethral rupture and two cases of a large albuginea tear (more than 2 cm.) with only a limited hematoma and slight penile swelling. Ultrasound examination can evidence the albuginea defect when the subcutaneous oedemaand the hematoma are limited. When a urethral rupture was suspected following urethral bleeding or an inability to urinate, then a preoperative flexible cistoscopy examination is mandatory and allows one to pose the correct diagnosis in all cases with minimal invasivity. Unlike urethrography, this manoeuvre can be performed during the operation with a sterile technique thus avoiding surgical field contamination and a waste of time.

We recommend immediate explorative surgery in the presence of evident physical signs of major haemorrhage and penile deformity because ultrasound diagnosis is more difficult in these patients due to the pronounced subcutaneous blood embedding and oedema. In these conditions a corporeal albuginea tear is present in 100% of patients.(**Table1**).

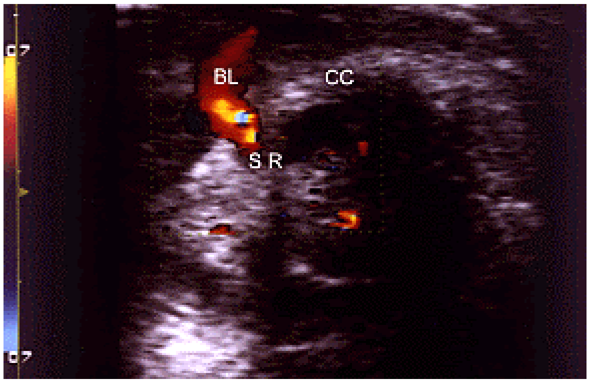

**Figure 8**

**TABLE 1**

| Pt | Age | Hemotoma | Voiding Problems | Elapsed Time | Cystoscopy | First Approach | Urethral lesion | Follow-up | Healing |
|---|---|---|---|---|---|---|---|---|---|
| 1 | 22 | severe | no | 2 hours | intraoperative | surgery | no | 18 months | ok |
| 2 | 20 | severe | no | 10 | intraoperative | surgery | no | 8 years | ok |
| 3 | 35 | severe | no | 6 | intraoperative | surgery | no | 28 months | ok |
| 4 | 31 | severe | blood from urethra | 48 | preoperative | surgery | yes | 12 months | curvature |
| 5 | 20 | light | yes | 12 | preoperative | ultrasound + cav | yes | 8 months | ok |
| 6 | 43 | light | no | 5 | no | cavernosography | no | 5 years | ok |
| 7 | 25 | light | no | 12 | no | ultrasound + cav | no | 12 months | ok |
| 8 | 30 | light | no | 24 | no | cavernosography | no | 2 months | curvature |

Prompt surgery gives better aesthetic and functional results, as evidenced by the two patients in our series with post-operative curvature who were operated after 24 and 48 hours. Experience with penile and urethral surgery is important when a lesion is severe or associated to urethral injury. In fact, surgical skills avoid postoperative complications such as shaft curvature, corporal narrowing or urethral strictures. When a corporal lesion is associated with urethral injury great care must be taken to avoid contact between the severed spongious tissue and corporal tissue in order to obviate the risk of post-operative impotence as a consequence of a spongio-cavernous fistula. For these reasons we think that a penile fracture does not require "emergency room" surgery but should be carefully evaluated and treated following strategies and tactics which are well known to experienced urologists.

### REFERENCES

1. Benet AE; Melman A: The epidemiology of erectile dysfunction.î Urol Clin North Am, 1995 Nov, 22:4, 699-709
2. Goldman HB; Dmochowski RR; Cox CE: Penetrating trauma to the penis: functional resultsî.J Urol, 1996 Feb, 155:2, 551-3
3. Ilkay AK; Levine LA: ìConservative management of high-flow priapism.î Urology, 1995 Sep, 46:3, 419-24
4. Lazinger M; Beckmann CF; Cossi A; Roth RA: Selective embolization of bilateral arterial cavernous fistulas for posttraumatic penile arterial priapismî. Cardiovasc Intervent Radiol, 1996 Jul-Aug, 19:4, 281-4
5. Lehmann K; Schöpke W; Hauri D: Subclinical trauma to perineum: a possible etiology of erectile dysfunction in young men. Eur Urol, 1995, 27:4, 306-10
6. Licht MR; Lewis RW; Sershon PD: Immediate impotence after penetrating perineal trauma: restoration of erections with penile artery revascularization, corpus cavernosum aneurysm repair, and deep penile venous ligationî. Urology, 1995 Oct, 46:4, 577-80
7. Matthews LA; Herbener TE; Seftel AD: Impotence associated with blunt pelvic and perineal trauma: penile revascularization as a treatment option ì. Semin Urol, 1995 Feb, 13:1, 66-72
8. Rahmouni A; Hoznek A; Duron A; Colombel M; Chopin DK; Mathieu D; Vasile N: Magnetic resonance imaging of penile rupture: aid to diagnosisî. J Urol, 1995 Jun, 153:6, 1927-8

**This article should be referenced as follows:**

De Stefani, S., Stubinski, R., Ferneti, F., Simonato, A., and Carmignani, G. (2004) Penile fracture and associated urethral injury. *TheScientificWorldJOURNAL* **4 (S1)**, 92–99.

**Handling Editor:**

Anthony Atala, Principle Editor for *Urology* — a domain of *TheScientificWorldJOURNAL*.
